# The Opioid Receptor Antagonist Naloxone Enhances First-Order Fear Conditioning, Second-Order Fear Conditioning and Sensory Preconditioning in Rats

**DOI:** 10.3389/fnbeh.2021.771767

**Published:** 2021-12-06

**Authors:** Robine M. L. Michalscheck, Dana M. Leidl, R. Frederick Westbrook, Nathan M. Holmes

**Affiliations:** School of Psychology, University of New South Wales, Sydney, NSW, Australia

**Keywords:** naloxone, pavlovian fear conditioning, second-order fear conditioning, sensory preconditioning, mediated conditioning, prediction error

## Abstract

The opioid receptor antagonist naloxone enhances Pavlovian fear conditioning when rats are exposed to pairings of an initially neutral stimulus, such as a tone, and a painful foot shock unconditioned stimulus (US; so-called first-order fear conditioning; Pavlov, 1927). The present series of experiments examined whether naloxone has the same effect when conditioning occurs in the absence of US exposure. In Experiments 1a and 1b, rats were exposed to tone-shock pairings in stage 1 (one trial per day for 4 days) and then to pairings of an initially neutral light with the already conditioned tone in stage 2 (one trial per day for 4 days). Experiment 1a confirmed that this training results in second-order fear of the light; and Experiment 1b showed that naloxone enhances this conditioning: rats injected with naloxone in stage 2 froze more than vehicle-injected controls when tested with the light alone (drug-free). In Experiments 2a and 2b, rats were exposed to light-tone pairings in stage 1 (one trial per day for 4 days) and then to tone-shock pairings in stage 2 (one trial per day for 2 days). Experiment 2a confirmed that this training results in sensory preconditioned fear of the light; and Experiment 2b showed that naloxone enhances sensory preconditioning when injected prior to each of the light-tone pairings: rats injected with naloxone in stage 1 froze more than vehicle-injected controls when tested with the light alone (drug-free). These results were taken to mean that naloxone enhances fear conditioning independently of its effect on US processing; and more generally, that opioids regulate the error-correction mechanisms that underlie associative formation.

## Introduction

One of the central ideas in the study of learning is that of error correction. The idea is that organisms compare a new experience with existing knowledge, evaluating the degree to which the experience is discrepant from what is already known. When the evaluation yields a discrepancy, knowledge is updated to bring it into line with the new experience. This idea originated in the demonstrations of blocking, contingency and signal validity effects reported in the classic experiments by Kamin (1968); Rescorla (1968), and Wagner et al. (1968), respectively. These experiments differed in several ways but were alike in showing that the normally effective relation for conditioning was rendered ineffective when the target conditioned stimulus (CS) was accompanied by a better predictor of the unconditioned stimulus (US). These results led to the Rescorla-Wagner model which held that conditioning was regulated by prediction error: by the difference between the amount that could be learned about the US and the amount that had already been learned by all the stimuli present (Rescorla and Wagner, 1972). This was formalized in the equation, ΔV = α×β × (λ - ΣV), where ΔV is the change in the strength of the association between a CS and US, α and β denote the effectiveness of the CS and US, respectively, and the bracketed term, λ - ΣV, reflects the discrepancy between the presence or absence of the US (λ) and its current expectancy (ΣV).

One of the many lines of investigation initiated by this model concerned the neural mechanisms that mediate error correction. The discovery of endogenous opioids (Hughes, 1975; Hughes et al., 1975; for review see McNally and Akil, 2002) and their activation by a CS paired with an aversive (e.g., shock) US led to the proposal that the error signal that regulates associative formation in Pavlovian fear conditioning is instantiated through the endogenous opioid system (Fanselow, 1984, 1998; for review see McNally, 2009). Evidence for this proposal was provided by demonstrations that a systemic injection of the opioid receptor agonist morphine given before CS-US presentations impairs the acquisition of fear to the CS; while a systemic injection of the opioid receptor antagonist naloxone before CS-US presentations enhances the acquisition of fear to the CS (e.g., Westbrook et al., 1991; McNally et al., 2004). An explanation for these findings is that opioid receptor agonists and antagonists alter the functional or perceived intensity of the shock US through opioid-mediated pain regulation (Madden et al., 1977; Harris, 1996). That is, opioid receptor agonists reduce pain sensitivity and US intensity (i.e., they decrease λ and hence the λ-ΣV quantity), thereby impairing fear acquisition; while opioid receptor antagonists enhance pain sensitivity and US intensity (they increase λ and hence the λ-ΣV quantity), thereby enhancing fear acquisition (e.g., Westbrook et al., 1991; Young and Fanselow, 1992).

However, naloxone has also been shown to affect the extinction of Pavlovian conditioned fear. McNally and Westbrook (2003) conditioned two groups of rats to fear a CS through its pairings with shock and then exposed them to a series of CS alone presentations to extinguish this fear. Rats received either a systemic injection of naloxone or vehicle before the CS alone presentations. Naloxone-treated rats exhibited an equivalent level of CS-elicited freezing as vehicle-controls at the start of the extinction session. However, unlike the controls, naloxone-treated rats failed to exhibit any significant decline in freezing across the first extinction session (i.e., they showed no evidence of within-session extinction learning) and exhibited a slower decline in freezing across subsequent extinction sessions. The contrasting effects of naloxone on the acquisition and extinction of conditioned fear suggests that opioid receptor activity does more than just affect the processing of the US. If naloxone only affected the processing of an aversive shock US (Fanselow and Bolles, 1979a,b), it should not have affected the extinction of conditioned fear which occurs in the absence of the US. Thus, in addition to regulating the functional intensity of the US, it has been suggested that naloxone reduces the contribution of prior conditioning experiences to the expectancy of an aversive event, and thereby, interferes with the error correction processes that underlie the acquisition and extinction of conditioned fear (McNally and Westbrook, 2006; for review see McNally, 2009). Expressed in terms of the Rescorla-Wagner model, naloxone may effectively block ΣV (i.e., ΣV remains zero), causing the discrepancy between λ - ΣV to persist for longer in acquisition, resulting in enhanced fear conditioning; and to diminish more rapidly in extinction, resulting in impaired fear extinction (for review see McNally, 2009; for further evidence in support of these ideas see Fanselow and Bolles, 1979a,b; McNally et al., 2004).

The proposal that naloxone acts on error-correction mechanisms implies that it may enhance fear conditioning independently of its effects on US processing (i.e., by maintaining ΣV at zero rather than by increasing λ). This implication can be tested through the use of protocols that produce fear conditioning in the absence of US exposure. One such protocol is second-order fear conditioning which is typically produced by first pairing a neutral stimulus (S1) with an aversive US and then pairing a second neutral stimulus (S2) with the already conditioned, fear-eliciting S1. As far as we are aware, only one previous study has examined the role of endogenous opioids in the acquisition of second-order conditioned fear. Cicala et al. (1990) injected rats with either naloxone or vehicle before second-order fear conditioning. At test, naloxone-injected rats exhibited more fear to S2 (as indexed by conditioned lick suppression) compared to vehicle-injected controls. However, the vehicle-injected rats in this experiment showed no evidence of having acquired second-order conditioned fear to S2: they exhibited as little fear of S2 as rats in control groups that received either unpaired presentations of S1 and the US in stage 1 or of S2 and S1 in stage 2. This leaves open the possibility that, rather than enhancing acquisition of second-order conditioned fear, naloxone simply altered the generalization of fear from S1 to S2. That is, the Cicala et al. (1990) study leaves open the question of whether naloxone enhances second-order fear conditioning, and more generally, whether naloxone facilitates the formation of associations between stimuli that are not innately aversive, as seen in second-order fear conditioning and sensory preconditioning.

The present study addressed this gap in knowledge. It had two specific aims. The first was to identify the effect of naloxone on the acquisition of second-order conditioned fear. To this end, Experiment 1a established a one-trial-per-day second-order conditioning protocol; and Experiment 1b used this protocol to assess the effect of naloxone on the acquisition of both first- and second-order conditioned fear. The second aim was to identify the effect of naloxone on the acquisition of sensory preconditioned fear. To this end, Experiment 2a established a one-trial-per-day sensory preconditioning protocol; and Experiment 2b used this protocol to assess the effect of naloxone on the acquisition of first-order and sensory preconditioned fear.

## Experiment 1A

The aim of this experiment was to demonstrate second-order conditioned fear using a protocol in which rats received a single conditioning trial each day across successive days. Such a protocol was used previously to show that rats given a single CS-US trial each day under a systemic injection of naloxone froze more across successive trials than control rats injected with vehicle (McNally et al., 2004). This protocol has the advantage of ensuring that the effects of naloxone are equivalent across every trial of conditioning: i.e., it alleviates any concern that the effects of naloxone may dissipate across a longer conditioning session that includes multiple trials. The successful demonstration of second-order conditioned fear in this protocol would then allow us to examine whether rats given a single second-order conditioning trial each day under naloxone would also freeze more across successive trials than vehicle-treated controls. The protocol involved exposing one group of rats (labeled PP) to a single pairing (P) of an auditory stimulus (S1) and foot shock each day across four successive days (stage 1) and, after extinction of any freezing elicited by the context, exposing them to a single pairing (P) of a visual stimulus (S2) and the conditioned S1 each day across four successive days (stage 2). Finally, rats were tested for levels of freezing elicited by S2. A second group (PU) was included to assess whether the test levels of freezing to S2 in Group PP were due to the associations produced by its pairings with the conditioned S1 rather than to generalization from the conditioned S1. Rats in this group were also exposed to a single S1-shock pairing each day in stage 1 and to single presentations of S2 and S1 each day in stage 2, but these presentations were unpaired. A final group (UP) was included to assess whether the levels of freezing elicited by S2 in Group PP were due to its pairings with the conditioned S1. Rats in this group received unpaired presentations of S1 and the shock US in stage 1 and daily pairings of S2 with S1 in stage 2 (see Table 1).

**TABLE 1:** Design of Experiments 1a, 1b, 2a and 2b.

Group	Stage 1	Stage 2	S2 test	S1 test
**Experiment 1a**				
PP	S1+	S2–S1	S2−	S1−
PU	S1+	S1/S2		
UP	+/S1	S2–S1		
**Experiment 1b**				
NAL-NAL	(NAL) S1+	(NAL) S2–S1	S2−	S1−
NAL-VEH	(NAL) S1+	(VEH) S2–S1		
VEH-NAL	(VEH) S1+	(NAL) S2–S1		
VEH-VEH	(VEH) S1+	(VEH) S2–S1		
**Experiment 2a**				
PP	S2–S1	S1+	S2−	S1−
PU	S2–S1	+/S1		
UP	S1/S2	S1+		
**Experiment 2b**				
NAL-NAL	(NAL) S2–S1	(NAL) S1+	S2−	S1−
VEH-NAL	(VEH) S2–S1	(NAL) S1+		
NAL-VEH	(NAL) S2–S1	(VEH) S1+		
VEH-VEH	(VEH) S2–S1	(VEH) S1+		

*A plus sign (+) following one event indicates that it was co-terminated with shock; a minus sign (**−**) between events indicates that they were paired; a forward-stroke sign (/) indicates that they were explicitly unpaired; and a minus sign (−) following one event indicates it was presented alone. NAL = a subcutaneous injection of naloxone (2.5 mg/ml) and VEH = a subcutaneous injection of vehicle only. All injections were administered 5 min before the start of the training/test session.*

### Materials and Methods

#### Subjects

Subjects were 23 (7 males, 16 females) experimentally naive, adult Long Evans rats (250–450 g) obtained from the breeding facility maintained by the School of Psychology at the University of New South Wales. The rats were housed by sex in plastic tubs (67 cm length × 40 cm width × 22 cm height) with 3–4 rats per tub. The tubs were kept in an air-conditioned colony room whose temperature was maintained at 20 degrees Celsius and whose lights were on between 07:00 and 19:00. All rats had *ad libitum* access to water and food throughout the experiment.

#### Apparatus

Training and testing occurred in a set of eight identical chambers (30 cm length × 26 cm width × 30 cm height). The front and rear walls of each chamber were clear Plexiglas, the side walls and ceiling were aluminum, and the floor was constructed of stainless-steel rods, each 7 mm in diameter and spaced 1.8 mm apart. A shock could be delivered through the rods via a custom-built generator located in another room in the laboratory. Each chamber was located in its own light- and sound-attenuating wooden cabinet. A 2 × 3 array of white LEDs, a speaker, and a camera were mounted on the back wall of each cabinet and an infrared light was mounted on its ceiling. The LEDs and the speaker were used to present the auditory and visual stimuli. The camera was connected to a monitor and DVD recorder that were located in another room in the laboratory and used to record the behavior of each rat.

#### Stimuli

The two stimuli were a 1,000 Hz, 72 dB tone and a 3 Hz, 57 lux flashing light measured at the center of each chamber. These stimuli were used as the S1 and S2 stimuli, respectively. Each presentation of S1 lasted for 10 s and each presentation of S2 lasted for 30 s. The US was a 0.8 mA 1 s foot shock. Stimuli were programmed and presented using MATLAB software.

#### Scoring

Freezing, defined as the absence of all movement except that required for breathing, was the measure of conditioning (Fanselow, 1980). A time sampling procedure was used in which each rat was observed once every 2 s and its behavior scored as “freezing” or “not freezing.” A percentage score was calculated to determine the proportion of total observations each rat spent freezing on each trial. All test data were scored by the experimenter and an experienced observer who was blind to the group allocations and purpose of the experiment. The Pearson product-moment correlation between the experimenter’s and observer’s scores was > 0.9 in all experiments. Any discrepancies between the experimenter’s and observer’s scores were resolved in favor of the blind observer.

#### Statistical Analyses

The principal data obtained in Experiment 1a were acquisition of freezing to S1 in stage 1, acquisition of freezing to S2 and retention of freezing to S1 in stage 2, and test levels of freezing to both S2 and S1. The freezing data for S1 and S2 were analyzed separately in acquisition and testing using mixed model ANOVAs with a between-subject factor of group (Groups PP, PU, and UP), and a within-subject factor of trial (in acquisition) or block-of-trials (in testing). For all statistical analyses, the criterion for rejection of the null hypothesis was set at alpha = 0.05. With 1 and 20 degrees of freedom (df), the F critical (*F_c_*) was 4.35. Partial eta-squared ($\eta_{p}^{2}$) was calculated as a measure of the effect size for all statistically significant differences ($\eta_{p}^{2}$ of 0.14 is considered a large effect size).

#### Procedure

On each of days 1–4 (stage 1), rats in Groups PP and PU received a single presentation of the 10 s S1 which co-terminated with the foot shock. The onset of S1 occurred 2 min after placement in the chamber and rats remained in the chambers for an additional 1 min after the foot shock. Rats in Group UP received the foot shock ∼10 s after placement in the chambers and S1 approximately 3 min later. They were then removed from the chamber a few seconds later. On each of days 5–8, all rats received a 20 min exposure to the chambers in the absence of any scheduled events. This was done to extinguish any freezing elicited by the chambers; freezing that would obscure the subsequent detection of second-order conditioning.

On each of days 9–12 (stage 2), rats in Groups PP and UP received a single presentation of the 30 s S2 which co-terminated in the onset of the 10 s S1. The onset of S2 occurred 4.5 min after placement into the chamber and rats remained in the context for an additional 2 min after offset of the S1. Rats in Group PU received a presentation of the 10 s S1 a few seconds after placement in the chambers and approximately 5.5 min later a presentation of the 30 s S2. They were removed from the chambers a few seconds later. On each of days 13 and 14, all rats were exposed to the chambers in the absence of any scheduled events to extinguish any freezing elicited by the context alone.

Rats were tested with S2 and S1 on days 15 and 16, respectively. There were eight presentations of the 30 s S2 and 16 presentations of the 10 s S1. We doubled the number of S1 presentations because short duration stimuli typically require a greater number of trials to extinguish and we wanted to avoid any potential ceiling effects in the test of the S1. Onset of the first stimulus presentation occurred 3 min after placement in the chambers, the interval between stimulus presentations was fixed at 3 min, and rats remained in the chambers for a further 2 min after the final stimulus presentation.

### Results

**FIGURE 1:**
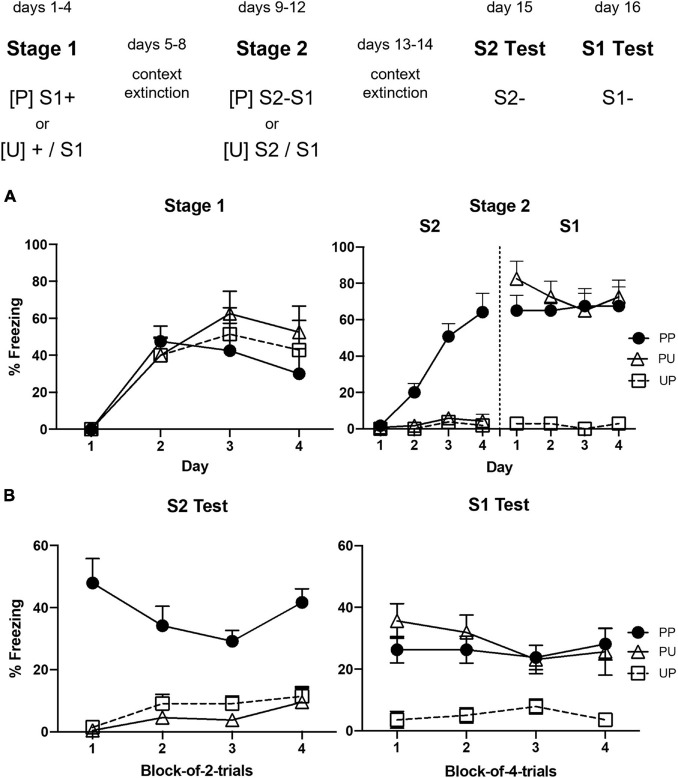
Results of Experiment 1a showing that freezing to S2 is due to second-order conditioning. **(A)** Shows the mean (+ SEM) levels of freezing to S1 in stage 1 (left panel) and to S2 and the previously conditioned S1 in stage 2 (right panel). **(B)** Shows the mean (+ SEM) levels of freezing during the final drug-free tests across blocks of two S2 alone trials (left panel) and four S1 alone trials (right panel). The numbers of rats in each group were: Group PP, *n* = 8; Group PU, *n* = 8; and Group UP, *n* = 7.

Figure 1A shows the mean levels of freezing to S1 across the 4 days of stage 1 (left panel) and the mean levels of freezing to S2 and S1 across the 4 days of stage 2 (right panel). They suggest that freezing increased in all groups across stage 1; however, only Groups PP and PU froze to S1 in stage 2 and only Group PP acquired freezing to S2. The statistical analyses supported these impressions. The analysis of freezing to S1 in stage 1 confirmed that there was a significant linear increase in freezing, *F*_(1, 20)_ = 19.27, *p* = 0.0003, $\eta_{p}^{2}$ = 0.49, CI [0.58, 1.63] and that there were no significant between-group differences in the rate of this increase or the overall levels of freezing, *F*s < 1. The analysis of freezing to S1 in stage 2 revealed that it remained stable across the S2–S1 pairings (no significant linear trend, *F* < 1) and there was no trend × group interaction, *F* < 1. However, there were between-group differences such that rats in Groups PP and PU that had received S1-shock pairings in stage 1 froze more to S1 than those in Group UP given unpaired presentations of S1 and shock in stage 1, *F*_(1, 20)_ = 59.03, *p* = 0.0001, $\eta_{p}^{2}$ = 0.75, CI [2.12, 3.71]. It is worth noting that the freezing by rats in UP across stage 1 likely reflected context conditioning which, of course, had been extinguished before stage 2, revealing the absence of conditioning to the unpaired S1. The statistical analysis of freezing to S2 revealed a significant linear increase, *F*_(1, 20)_ = 37.40, *p* < 0.0001, $\eta_{p}^{2}$ = 0.65, CI [0.99, 2.02] and a significant trend × group interaction, *F*_(1, 20)_ = 55.72, *p* < 0.0001, $\eta_{p}^{2}$ = 0.74, CI [2.78, 4.94], which, from inspection of the figure, was due to the increase in freezing by rats in Group PP. Finally, rats in this group froze significantly more to S2 than those in Groups PU and UP, *F*_(1, 20)_ = 58.35, *p* < 0.0001, $\eta_{p}^{2}$ = 0.75, CI [1.74, 3.04].

Figure 1B shows the mean levels of freezing in each group during the drug-free tests of S2 (left panel) and S1 (right panel). They suggest that Group PP froze more to S2 than Groups PU and UP, and that Groups PU and PP froze more to S1 than Group UP. The statistical analysis again supported these impressions. The analysis of freezing to S2 confirmed that Group PP froze significantly more to S2 than Groups PU and UP, *F*_(1, 20)_ = 75.40, *p* < 0.0001, $\eta_{p}^{2}$ = 0.79, CI [1.51, 2.47]. There was no statistically significant linear trend in freezing across the S2 alone presentations or trend × group interaction (Figure 1B, left panel), largest *F* < 4. The analysis of freezing to S1 confirmed that Groups PP and PU froze significantly more to S1 than Group UP, *F*_(1, 20)_ = 10.88, *p* < 0.0036, $\eta_{p}^{2}$ = 0.35, CI [0.43, 1.90]. There was no significant linear trend in freezing across the S1 alone presentations or trend × group interactions (Figure 1B, right panel), *F*s < 1.

### Discussion

This experiment has shown that rats exposed to S1-shock pairings in stage 1 and then to S2–S1 pairings in stage 2 (Group PP) froze more when tested with S2 than rats in two control groups: one exposed to S1-shock pairings but unpaired presentations of S2 and S1 (Group PU), and the other exposed to unpaired presentations of S1 and shock but S2–S1 pairings (for similar demonstrations, see Rizley and Rescorla, 1972; Yin et al., 1994; Parkes and Westbrook, 2010; Witnauer and Miller, 2011; Holmes et al., 2013). Thus, these results show that freezing to S2 in Group PP was associatively mediated, due to the S1-shock and S2–S1 pairings rather than generalization from the conditioned S1 or to any unconditioned ability of S1 to condition freezing to S2. Critically, this demonstration of second-order conditioned fear was obtained in the single trial per day protocol previously used to demonstrate that naloxone enhances first-order conditioned fear (McNally et al., 2004). The next experiment used this protocol to assess whether naloxone also enhances second-order conditioned fear.

## Experiment 1B

This experiment had two aims. The first was to replicate previously reported findings that naloxone enhances acquisition of first-order conditioned fear (McNally et al., 2004). The second aim was to determine the effect of naloxone on acquisition of second-order conditioned fear. The conditioning protocol was the same as that used for Group PP in the previous experiment: rats received a single S1-shock pairing on each of days 1–4, context alone exposures (to extinguish context-elicited freezing) across days 5–8, and a single S2–S1 pairing on each of days 9–12. Two groups received an injection of naloxone prior to each of the S1-shock pairings in stage 1 (Groups NAL-VEH and NAL-NAL), while the remaining two groups received an injection of vehicle only prior to these pairings (Groups VEH-NAL and VEH-VEH). One group in each of these pairs received an injection of naloxone prior to each of the S2–S1 pairings in stage 2 (Group NAL-NAL and VEH-NAL), while the other received an injection of vehicle only prior to these pairings (Groups NAL-VEH and VEH-VEH). Finally, all rats received extinction of any context-elicited freezing on days 13 and 14; and were tested with S2 on day 15 and S1 on day 16 (see Table 1).

We expected to replicate previous findings that naloxone enhances acquisition of first-order conditioned fear: that is, we expected rats injected with naloxone prior to each of the single S1-shock pairings (Groups NAL-VEH and NAL-NAL) to exhibit faster acquisition of freezing to S1 as well as higher levels of freezing to S1 across subsequent second-order conditioning and testing. The second question of interest concerned the effect of naloxone on acquisition of second-order conditioned fear. If naloxone enhances fear conditioning independently of its effect on an aversive US, then rats that received naloxone injections prior to the S2–S1 pairings in stage 2 (Groups VEH-NAL and NAL-NAL) will freeze more to S2 than vehicle-treated rats (Groups NAL-VEH and VEH-VEH) across its pairings with S1 and on the subsequent drug-free S2 alone test.

### Materials and Methods

#### Subjects and Apparatus

Subjects were 32 (17 males, 15 females) experimentally naive, adult Long Evans rats (250–450 g). They were sourced, housed and handled as described for Experiment 1a. The apparatus and stimuli were those used in Experiment 1a.

#### Drugs

Naloxone hydrochloride (Sigma Aldrich, Sydney, Australia) was dissolved in 0.9% (wt/vol) non-pyrogenic saline to obtain a concentration of 2.5 mg/ml (McNally and Westbrook, 2003). Non-pyrogenic saline was also used for control injections (i.e., vehicle). All injections were administered subcutaneously (s.c.) into the dorsal neck region at a volume of 1 ml/kg. Past research that used this same dose and route of administration did not report any non-specific effects of naloxone on freezing or locomotor activity in rats (e.g., Fanselow and Bolles, 1979a; McNally and Westbrook, 2003).

#### Scoring and Statistical Analyses

The method of scoring was identical to that described in Experiment 1a. The principal data were acquisition of freezing to S1 in stage 1, acquisition of freezing to S2 and retention of freezing to S1 in stage 2, and test levels of freezing to S2 and S1. The data for S1 and S2 were analyzed separately in acquisition and testing using a mixed model ANOVA with between-subject factors of stage 1 treatment (naloxone or vehicle) and stage 2 treatment (naloxone or vehicle); and a within-subject factor of trial (in acquisition) or block-of-trials (in testing). For all analyses, the criterion for rejection of the null hypothesis was set at alpha = 0.05. With 1 and 28 df, this yielded an *F_c_* of 4.2. Partial eta-squared ($\eta_{p}^{2}$) was calculated as a measure of the effect size for all statistically significant differences ($\eta_{p}^{2}$ of 0.14 is considered a large effect size).

#### Procedure

On each of days 1–4 (stage 1), rats received an injection of naloxone (Groups NAL) or vehicle (VEH). Five min later, they were placed in the conditioning chambers and exposed to a single S1-shock pairing in the manner described for Group PP in Experiment 1a. On each of days 5–8, all rats received an injection of vehicle and, 5 min later, were placed in the context for one 20 min session of context extinction. These sessions were intended to extinguish any freezing elicited by the chambers prior to the S2–S1 pairings in stage 2.

On each of days 9–12 (stage 2), rats received an injection of naloxone (Groups NAL-NAL and VEH-NAL) or vehicle (Groups NAL-VEH and VEH-VEH). Five min later, they were placed in the chambers and exposed to a single S2–S1 pairing in the manner described for Group PP in Experiment 1a. On each of days 13 and 14, all rats received an injection of vehicle and, after 5 min, were placed in the chambers for 20 min in the absence of any scheduled events. This was done to extinguish any such freezing that could obscure detection of the freezing elicited across the testing of S2 and S1.

On days 15 and 16, all rats received an injection of vehicle and, 5 min later, were tested for levels of freezing to S2 (day 15) and S1 (day 16). The details for these test sessions were identical to those described for Experiment 1a.

### Results

**FIGURE 2:**
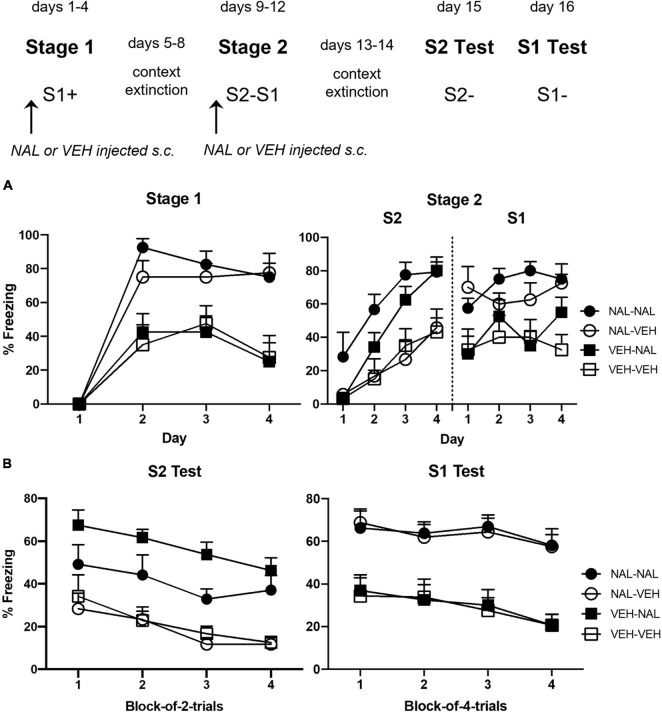
Results of Experiment 1b showing that naloxone enhances the acquisition of first- and second-order conditioned fear. **(A)** Shows the mean (+ SEM) levels of freezing to S1 in stage 1 (left panel) and to S2 and the previously conditioned S1 in stage 2 (right panel). **(B)** Shows the mean (+ SEM) levels of freezing during the final drug-free tests across blocks of two S2 alone trials (left panel) and four S1 alone trials (right panel). The numbers of rats in each group were: Group NAL-NAL, *n* = 8; Group NAL-VEH, *n* = 8; Group VEH-NAL, *n* = 8; and Group VEH-VEH, *n* = 8.

Figure 2A shows the mean levels of freezing to S1 across its pairings with shock in stage 1 (left panel) and to S2 and S1 across their pairings in stage 2 (right panel). It suggests that naloxone enhanced acquisition of both forms of conditioning but did not affect retrieval/expression of the already conditioned fear to S1. These impressions were confirmed by the statistical analyses. During stage 1, averaged across all groups, there was a significant linear increase in freezing across the daily S1-shock pairings, *F*_(1,__28)_ = 71.91, *p* < 0.0001, $\eta_{p}^{2}$ = 0.72, CI [1.19, 1.94]. The rate of this increase differed between the naloxone- and vehicle-treated groups, *F*_(1,__28)_ = 14.52, *p* = 0.0007, $\eta_{p}^{2}$ = 0.34, CI [0.65, 2.16]. Groups NAL-VEH and NAL-NAL acquired freezing more rapidly and froze more to S1 than Groups VEH-VEH and VEH-NAL, *F*_(1,__28)_ = 29.39, *p* < 0.0001, $\eta_{p}^{2}$ = 0.51, CI [0.81, 1.80]. The remaining main effects and interactions were not statistically significant, *F*s < 1.

The analysis of freezing to S2 across its pairings with S1 revealed a similar pattern of results. Averaged across all groups, there was a significant linear increase in freezing to S2, [*F*_(1,__28)_ = 125.68, *p* < 0.0001], $\eta_{p}^{2}$ = 0.82, CI [1.48, 2.14]. The rate of this increase differed between groups injected with naloxone or vehicle, *F*_(1,__28)_ = 6.67, *p* = 0.0153, $\eta_{p}^{2}$ = 0.19, CI [0.17, 1.49]. Groups VEH-NAL and NAL-NAL acquired freezing more rapidly and froze more to S2 than Groups VEH-VEH and NAL-VEH, *F*_(1,__28)_ = 18.10, *p* = 0.0002, $\eta_{p}^{2}$ = 0.39, CI [0.61, 1.75]. The remaining main effects and interactions were not statistically significant, largest *F* < 3. The analysis of freezing to the conditioned S1 revealed no significant linear trend, *F* < 4; and no significant trend × group interactions, largest *F* < 3. The overall level of freezing to S1 did not differ between groups exposed to the S2–S1 pairings under naloxone or vehicle, *F* < 1, but did differ between groups that had been injected with naloxone or vehicle across the prior S1-shock pairings: those that received naloxone in stage 1 (Groups NAL-VEH and NAL-NAL) froze more to S1 than those that received vehicle in stage 1 (Groups VEH-NAL and VEH-VEH), *F*_(1,__28)_ = 14.68, *p* = 0.0007, $\eta_{p}^{2}$ = 0.34, CI [0.51, 1.69].

Figure 2B shows the mean levels of freezing in each group during drug-free testing with S2 (left panel) and S1 (right panel). It suggests that rats that had been injected with naloxone prior to each of the S2–S1 pairings (Groups VEH-NAL and NAL-NAL) froze more to S2 than rats that had been injected with vehicle before these pairings (Groups VEH-VEH and NAL-VEH); and rats that had been injected with naloxone prior to each S1-shock pairing (Groups NAL-VEH and NAL-NAL) froze more to S1 than rats that had been injected with vehicle (Groups VEH-VEH and VEH-NAL) before these pairings. These impressions were confirmed by the statistical analyses. In the S2 test, averaged across all groups, there was a significant linear decline in freezing across the S2 alone presentations, *F*_(1,__28)_ = 20.31, *p* = 0.0001, $\eta_{p}^{2}$ = 0.42, CI [−1.29, −0.49]. Overall, groups injected with naloxone prior to each of the daily S2–S1 pairings (VEH-NAL and NAL-NAL) froze more to S2 than groups injected with vehicle (VEH-VEH and NAL-VEH), *F*_(1,__28)_ = 45.70, *p* < 0.0001, $\eta_{p}^{2}$ = 0.62, CI [1.14, 2.12]. Moreover, groups injected with naloxone prior to each of the daily S1-shock pairings (NAL-VEH and NAL-NAL) froze less to S2 than groups injected with vehicle (VEH-NAL and VEH-VEH) before these pairings, *F*_(1,__28)_ = 5.08, *p* = 0.0322, $\eta_{p}^{2}$ = 0.15, CI [−1.04, −0.05]. There was no significant difference in freezing to S2 between Groups NAL-VEH and VEH-VEH (*F* < 1), indicating that the naloxone injections prior to S1-shock pairings did not automatically increase second-order freezing to S2. There were no significant trend × group interactions, largest *F* < 3. In the S1 test, averaged across all groups, there was a significant linear decline in freezing across the S1 alone presentations, *F*_(1,__28)_ = 7.81, *p* = 0.0093, $\eta_{p}^{2}$ = 0.22, CI [−0.80, −0.12]. Overall, groups injected with naloxone prior to the S1-shock pairings (NAL-NAL and NAL-VEH) froze more to S1 than groups injected with vehicle (VEH-NAL and VEH-VEH), *F*_(1,__28)_ = 43.19, *p* < 0.0001, $\eta_{p}^{2}$ = 0.61, CI [1.35, 2.57]. However, there was no significant difference in freezing to S1 between rats that had been injected with naloxone or vehicle in stage 2, and no significant interactions between linear trend and groups, *Fs* < 1.

### Discussion

This experiment has revealed three major findings. First, naloxone acutely enhanced the acquisition of first-order fear to S1 and second-order fear to S2: rats injected with naloxone prior to each S1-shock pairing in stage 1 (administered one per day) froze more to S1 than rats injected with vehicle; and rats injected with naloxone prior to each S2–S1 pairing in stage 2 (again administered one per day) froze more to S2 than rats injected with vehicle. Second, during the S2–S1 pairings in stage 2, freezing to the already conditioned S1 was unaffected by the naloxone injection: that is, rats injected with naloxone prior to each S2–S1 pairing in stage 2 froze to S1 at the same level as rats injected with vehicle. Finally, in the drug-free tests of S2 and S1, the enhancing effect of naloxone on first- and second-order fear conditioning persisted such that rats that had received naloxone in stage 2 froze more to S2 than rats that had received vehicle in stage 2; and rats that had received naloxone in stage 1 froze more to S1 than rats that had received vehicle in stage 1. The implication of these findings will be explored in section “General Discussion.”

## Experiment 2A

The aim of this experiment was to demonstrate sensory preconditioned fear using a one-trial-per-day protocol that could then be used to assess the effect of naloxone on that form of learning. The design was the same as that used in Experiment 1a, except that the order of the training stages was reversed (see Table 1). Rats in Group PP were exposed to a single S2–S1 pairing each day in stage 1 and then to a single S1-shock pairing each day in stage 2; rats in Group PU were exposed to a single S2–S1 pairing each day in stage 1 but to an unpaired presentation of S1 and shock each day in stage 2; and, finally, rats in Group UP were exposed to unpaired presentations of S2 and S1 each day in stage 1 but to a single S1-shock pairing each day in stage 2. Finally, all rats were tested for freezing to S2 and S1. The rationale for such a design was that described previously. To show that any freezing elicited by S2 in Group PP was due to the associations produced by the pairings in each stage, it was necessary to assess whether: the pairings of S2 and S1 in stage 1 were sufficient to imbue S2 with the ability to elicit freezing in the absence of any fear conditioning of S1 (Group PU); and the degree to which freezing conditioned to S1 generalized to S2 (Group UP).

### Materials and Methods

#### Subjects and Apparatus

Subjects were 25 (9 males, 16 females) experimentally naive, adult Long Evans rats (250–450 g). They were sourced, housed and handled as described in Experiment 1a. The stimuli and apparatus were the same as those used in previous experiments.

#### Scoring and Statistical Analyses

The method of scoring was identical to that used in previous experiments. The principal data obtained were acquisition of freezing to S1 in stage 2 and test levels of freezing to S2 and S1. The data for S1 and S2 were analyzed separately using mixed model ANOVAs with a between-subject factor of group (PP, PU, and UP) and a within-subject factor of trial (in acquisition) or block-of-trials (in testing). For all analyses, the criterion for rejection of the null hypothesis was set at alpha = 0.05. With 1 and 22 df, this yielded a ***F*_c_** of 4.30. Partial eta-squared (**$\eta_{p}^{2}$**) was calculated as a measure of the effect size for all statistically significant differences.

#### Procedure

On each of days 1–4 (stage 1), rats in Groups PP and PU were placed in the chambers and 4.5 min later exposed to a 30 s S2 which co-terminated in the onset of the 10 S1. They were removed from the chambers 2 min later. Rats in Group UP were exposed to the 10 s S1 a few seconds after placement in the chambers and 5.5 min later to the 30 s S2. They were removed from the chambers a few seconds later.

On each of days 5 and 6 (stage 2), rats in Groups PP and UP received a single presentation of the 10 s S1 which co-terminated with the 1 s foot shock. We reduced the number of S1-shock pairings (two in total) relative to the number used in Experiments 1a and 1b (four) as we wanted to increase the sensitivity of the sensory preconditioning protocol to any potential effect of naloxone. The onset of S1 occurred 2 min after placement in the chamber and rats remained in the chambers for an additional 1 min. On each of these days, rats in Group PU were shocked a few seconds after placement in the chambers, presented with S1 3 min later, and removed from the chambers a few seconds later. On each of days 7 and 8, all rats were exposed to the chambers for 20 min in the absence of any scheduled events to extinguish any freezing elicited by the chambers; freezing that would obscure detection of the freezing elicited by S2 and S1.

On day 9, rats were tested with S2 and on day 10 with S1. Testing consisted in 16 S2 alone presentations, each 30 s, and 16 S1 alone presentations, each 10 s. The first stimulus presentation occurred 3 min after placement in the chambers, the interval between presentations was fixed at 3 min, and rats remained in the chambers for 2 min after the final stimulus presentation.

### Results

**FIGURE 3:**
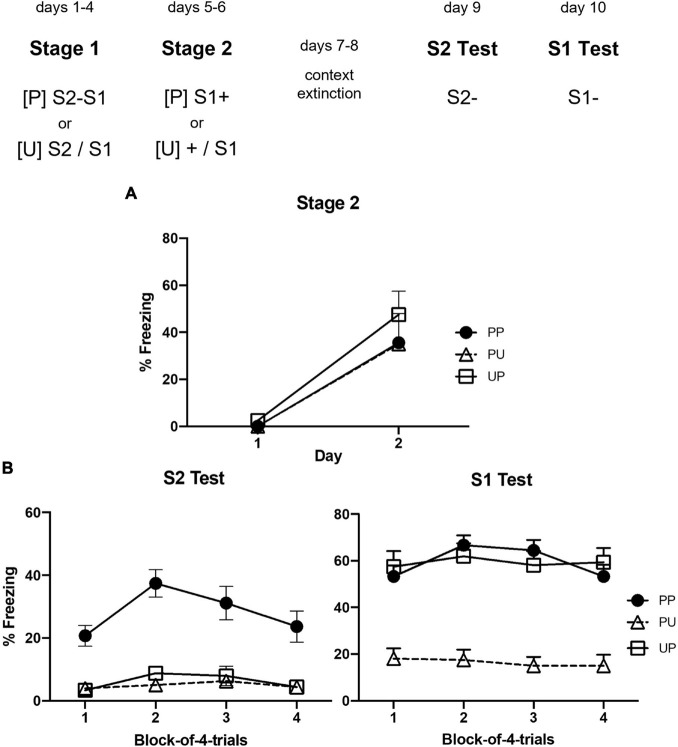
Results of Experiment 2a showing that freezing to S2 is due to sensory preconditioning. **(A)** Shows the mean (+ SEM) levels of freezing to S1 in stage 2. **(B)** Shows the mean (+ SEM) levels of freezing during the final drug-free tests across blocks of four S2 alone trials (left panel) and four S1 alone trials (right panel). The numbers of rats in each group were: Group PP, *n* = 9; Group PU, *n* = 8; and Group UP, *n* = 8.

Figure 3A shows the mean levels of freezing to S1 across sessions in which it was presented with shock in stage 2. Inspection of the figure indicates little or no freezing during the first presentation of S1 but substantial freezing in all groups during its second presentation. The statistical analysis confirmed that there was a significant increase in freezing to S1 across the two trials, *F*_(1, 22)_ = 22.50, *p* = 0.00098, $\eta_{p}^{2}$ = 0.51, CI [0.63, 1.61], but no significant trend × group interaction or between-group differences in the overall levels of freezing, largest *F* < 3.

Figure 3B shows the mean levels of freezing in each group during the tests of S2 (left panel) and S1 (right panel). It suggests that rats in Group PP froze more to S2 than rats in Groups PU and UP; and that rats in Groups UP and PP froze more to S1 than rats in Group PU. These impressions were confirmed by the statistical analyses. Group PP froze significantly more to S2 than Groups PU and UP, *F*_(1, 22)_ = 114.47, *p* < 0.0001, $\eta_{p}^{2}$ = 0.84, CI [1.68, 2.49], who did not differ from each other, *F* < 1. Averaged across all groups, there was no significant linear decline in freezing to S2 or significant interaction between linear trend and grouping, *F*s < 1. Groups PP and UP did not differ from each other, *F* < 1, but froze significantly more to S1 than Group UP, *F*_(1, 22)_ = 27.11, *p* < 0.0032, $\eta_{p}^{2}$ = 0.55, CI [1.12, 2.61]. Averaged across all groups, there was no significant linear decline in freezing to S1 or significant interaction between linear trend and grouping, *F*s < 1.

### Discussion

This experiment exposed rats in Group PP to a single S2–S1 pairing each day and then to a single S1-shock pairing each day. It found that these rats froze more when tested with S2 than control rats exposed to either S2–S1 pairings but unpaired presentations of S1 and shock (Group PU) or to unpaired presentations of S2 and S1 but pairings of S1 and shock (Group UP). These results show that a single pairing each day produces an association between S2 and S1 in stage 1; that a single pairing each day produces an association between S1 and shock in stage 2; and that the integration of these associations results in freezing when rats are tested with S2. The next experiment used this protocol to assess the effect of naloxone on the acquisition of sensory preconditioned fear.

## Experiment 2B

This experiment had two aims. The first was to replicate the finding that naloxone enhances acquisition of first-order conditioned fear (Experiment 1b; McNally et al., 2004). The second aim was to determine whether naloxone enhances the acquisition of sensory preconditioned fear just as it enhanced the acquisition of second-order conditioned fear (Experiment 1b). The protocol was the same as that used for Group PP in Experiment 2a (see Table 1). Rats in two groups received an injection of naloxone prior to each S2–S1 pairing in stage 1 (Groups NAL-VEH and NAL-NAL), while rats in another two groups received an injection of vehicle prior to each of these pairings (Groups VEH-NAL and VEH-VEH). One group in each pair then received an injection of naloxone prior to each S1-shock pairing in stage 2 (Groups VEH-NAL and NAL-NAL), while the other group received an injection of vehicle only prior to these pairings (Groups NAL-VEH and VEH-VEH). Finally, all rats were injected with vehicle and tested with S2 and then with S1. The questions of interest concerned the levels of freezing among rats that received naloxone relative to those that received vehicle prior to stage 1 and stage 2. We expected that naloxone would enhance the acquisition of first-order conditioned fear and, hence, that rats in Groups NAL-NAL and VEH-NAL would freeze more to S1 across its acquisition and testing than Groups VEH-VEH and NAL-VEH. If naloxone also enhanced the acquisition of the S2–S1 association in stage 1, then Groups NAL-VEH and NAL-NAL would freeze more to S2 across its testing than Groups VEH-NAL and VEH-VEH.

### Materials and Methods

#### Subjects and Apparatus

Subjects were 32 female, experimentally naive, adult Long Evans rats (250–300 g), sourced, housed and handled as described for Experiment 1a. The apparatus and stimuli were the same as those used in previous experiments. The details for drug/vehicle preparation and administration were the same as used in Experiment 1b.

#### Scoring and Statistical Analyses

The method of scoring was identical to that used in previous experiments. The principal data were acquisition of freezing to S1 in stage 2 and test levels of freezing to S2 and S1. The test data for S1 and S2 were analyzed separately using a mixed model ANOVA with between-subject factors of stage 1 treatment (naloxone or vehicle) and stage 2 treatment (naloxone or vehicle), and a within-subject factor of trial (in acquisition) or block-of-trials (in testing). For all analyses, the criterion for rejection of the null hypothesis was set at alpha = 0.05. With 1 and 28 df, this yielded a *F_c_* of 4.20. Partial eta-squared ($\eta_{p}^{2}$) was calculated as a measure of the effect size for all statistically significant differences.

#### Procedure

On each of days 1–4 (stage 1), rats received an injection of either naloxone or vehicle. Five minutes later, they were placed in the chambers and exposed to a single S2–S1 pairing in the manner described for Group PP in Experiment 2a.

On each of days 5 and 6 (stage 2), half of the rats that had been injected with naloxone in stage 1 were again injected with naloxone (Group NAL-NAL), while the remainder were injected with vehicle (Group NAL-VEH). Similarly, half of the rats that had been injected with vehicle in stage 1 were now injected with naloxone (Group VEH-NAL), while the remainder were again injected with vehicle (Group VEH-NAL). Five min after the injection, rats were placed in the chambers and exposed to a single S1-shock pairing in the manner described for Group PP in Experiment 2a. On each of days 7 and 8, all rats received an injection of vehicle and, after 5 min, were placed in the chambers for 20 min in the absence of any scheduled events. This was done to extinguish any freezing elicited by the chambers.

On days 9 and 10, rats were tested with S2 and S1, respectively. On each day, they received an injection of vehicle and, 5 min later, were placed in the chambers where they were tested with S2 (day 9) or S1 (day 10) in the manner described for Experiment 2a.

### Results

**FIGURE 4:**
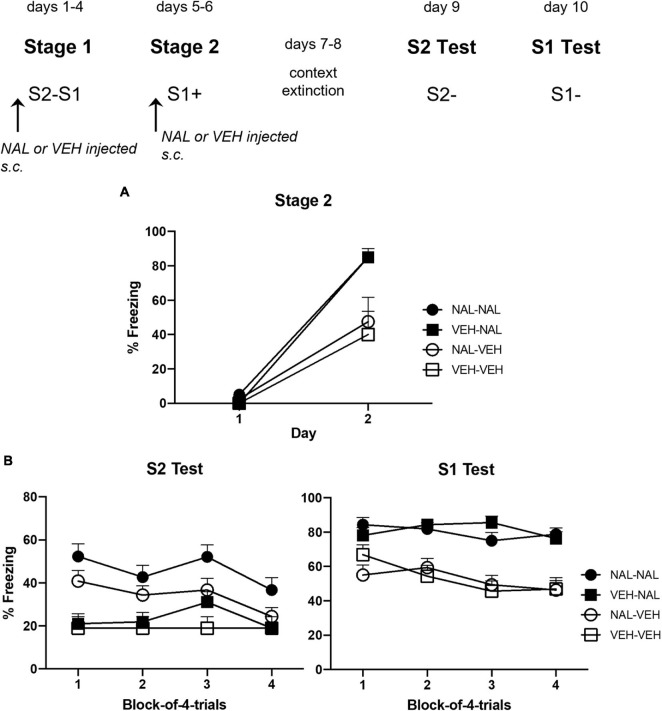
Results of Experiment 2b showing that naloxone enhances the acquisition of sensory preconditioning and first-order conditioned fear. **(A)** Shows the mean (+ SEM) levels of freezing to S1 across its pairings with shock in stage 2. **(B)** Shows the mean (+ SEM) levels of freezing during the final drug-free tests across blocks of four S2 alone trials (left panel) and four S1 alone trials (right panel). The numbers of rats in each group were: Group NAL-NAL, *n* = 8; Group VEH-NAL, *n* = 8; Group NAL-VEH, *n* = 8; and Group VEH-VEH, *n* = 8.

Figure 4A shows the mean level of freezing to S1 across its parings with shock in each of the four groups. The statistical analysis confirmed that naloxone enhanced conditioning. There was a significant linear increase in freezing across the pairings, *F*_(1,__28)_ = 130.60, *p* < 0.0001, $\eta_{p}^{2}$ = 0.82, CI [2.41, 3.47], and a significant trend × drug interaction, *F*_(1,__28)_ = 13.37, *p* = 0.0011, $\eta_{p}^{2}$ = 0.32, CI [0.83, 2.94]. Importantly, there was a significant drug effect such that rats injected with naloxone prior to each S1-shock pairing (Groups VEH-NAL and NAL-NAL) froze more to S1 than rats injected with vehicle (Groups VEH-VEH and NAL-VEH), *F*_(1,__28)_ = 17.00, *p* < 0.0003, $\eta_{p}^{2}$ = 0.38, CI [0.50, 1.50]. There was no significant interaction between the treatments in stages 1 and 2: naloxone or vehicle treatment in stage 1 did not affect freezing to S1 among naloxone- or vehicle-treated rats in stage 2 (*F*s < 1).

Figure 4B shows the mean levels of freezing in each group during drug-free testing with S2 (left panel) and S1 (right panel). Inspection of the left panel suggests that rats that had been injected with naloxone prior to each of the S2–S1 pairings in stage 1 (Groups NAL-NAL and NAL-VEH) froze more to S2 than rats injected with vehicle (Groups VEH-VEH and VEH-NAL). Inspection of the right panel suggests that rats injected with naloxone prior to each of the S1-shock pairings in stage 2 froze more to S1 (Groups VEH-NAL and NAL-NAL) than rats that had been injected with vehicle before these pairings (Groups VEH-VEH and NAL-VEH). The statistical analyses confirmed these impressions. The analysis of the S2 test data revealed a significant linear decline in freezing across the stimulus presentations, *F*_(1,__28)_ = 6.27, *p* = 0.0184, $\eta_{p}^{2}$ = 0.18, CI [−0.09, −0.94]. It also showed that, overall, groups injected with naloxone in stage 1 (NAL-VEH and NAL-NAL) froze more to S2 than groups injected with vehicle in stage 1 (VEH-VEH and VEH-NAL), *F*_(1,__28)_ = 14.58, *p* = 0.0007, $\eta_{p}^{2}$ = 0.34, CI [0.42, 1.41]. There was no significant difference in the level of freezing between rats that had been injected with naloxone or vehicle in stage 2, including between Groups VEH-NAL and VEH-VEH, *F* < 1, indicating that the naloxone injections prior to S1-shock pairings did not automatically increase sensory preconditioned freezing to S2. There was no significant interaction between linear trend and grouping, *F*s < 1.

The analysis of the S1 test data also revealed a significant linear decline in freezing across the stimulus presentations, *F*_(1,__28)_ = 6.85, *p* = 0.0144, $\eta_{p}^{2}$ = 0.20, CI [−0.64, −0.08]. Overall, groups injected with naloxone in stage 2 (NAL-NAL and VEH-NAL) froze more to S1 than groups injected with vehicle in stage 2 (NAL-VEH and VEH-VEH), *F*_(1,__28)_ = 14.42, *p* < 0.0007, $\eta_{p}^{2}$ = 0.34, CI [0.55, 1.83]. However, there was no significant difference in the level of freezing between rats that had been injected with naloxone or vehicle in stage 1, and no significant interactions between linear trend and grouping, largest *F* < 3.

### Discussion

This experiment has again confirmed that naloxone enhances the acquisition of first-order fear to S1. Rats injected with naloxone prior to the single S1-shock pairing on each of days 5 and 6 froze more to S1 on day 6 and across subsequent drug-free testing than rats conditioned under vehicle. It has also shown for the first time that naloxone enhances the acquisition of sensory preconditioned fear to S2: rats injected with naloxone prior to each S2–S1 pairing in stage 1 froze more when tested drug-free with S2 than rats injected with vehicle prior to these pairings. Importantly, the effects of naloxone on first-order fear to S1 and sensory preconditioning to S2 did not interact: in the final drug-free tests, rats that had received naloxone in stage 2 froze more to S1 than rats that had received vehicle in stage 2, regardless of the injection that rats had received in stage 1; and rats that had received naloxone in stage 1 froze more to S2 than rats that had received vehicle in stage 1, regardless of the injection that rats had received in stage 2. The implication of these findings will be explored in the section “General Discussion”.

## General Discussion

This series of experiments examined whether naloxone can enhance conditioning independently of its effect on US processing. It did so by examining the effect of naloxone on two forms of conditioning that occur in the absence of US exposure: second-order fear conditioning and sensory preconditioning. The initial experiments examined second-order conditioning. Experiment 1a established second-order conditioned fear using a protocol in which rats were exposed to a single S1-shock pairing on each day of stage 1 and a single S2–S1 pairing on each day of stage 2. Rats trained in this way froze more when tested with S2 alone than controls that had been exposed to explicitly unpaired presentations of the relevant stimuli in training, confirming that the freezing to S2 is due to second-order conditioning. Experiment 1b then used this one-trial-per-day protocol to assess the effect of naloxone on both first- and second-order conditioned fear. Relative to vehicle-injected controls, rats injected with naloxone prior to each S1-shock pairing exhibited faster acquisition of freezing to S1 and more freezing when it was tested drug-free; similarly, rats injected with naloxone prior to each S2–S1 pairing exhibited faster acquisition of freezing to S2 and more freezing when it was tested drug-free. Thus, naloxone enhances second-order fear conditioning just as it enhances first-order fear conditioning, thereby showing that it influences Pavlovian fear conditioning independently of its effect on US processing (e.g., Young and Fanselow, 1992): i.e., it enhances fear conditioning to a stimulus paired with danger regardless of whether the source of the danger is an aversive US, as in first-order conditioning, or a learned source of danger, as in second-order conditioning.

The remaining experiments examined whether the effects of naloxone on Pavlovian conditioning are specific to learning about danger. They did so by examining whether naloxone also enhances sensory preconditioning. Experiment 2a established sensory preconditioned fear using a protocol in which rats were exposed to a single S2–S1 pairing on each day of stage 1 and a single S1-shock pairing on each day of stage 2. Rats trained in this way froze more when tested with S2 alone than controls that had received explicitly unpaired presentations of the relevant stimuli in training, confirming that the freezing to S2 was associative in nature, due to the pairings of S2 and S1 in stage 1 and of S1 and foot shock in stage 2. Experiment 2b then used this one-trial-per-day protocol to assess the effect of naloxone on both first-order conditioned fear and sensory preconditioned fear. It replicated the finding that naloxone enhances first-order fear to S1 and showed, for the first time, that naloxone also enhances sensory preconditioning: relative to vehicle-injected controls, rats injected with naloxone prior to each S2–S1 pairing exhibited more freezing to S2 when it was tested drug-free. These results show that the effects of naloxone are not specific to learning about danger: rather, naloxone enhances associative formation between stimuli that are presented together, including associative formation between neutral stimuli in sensory preconditioning.

The common effect of naloxone on the different types of conditioning suggests that, just as opioids encode the error signal that underlies first-order fear conditioning (e.g., McNally and Westbrook, 2006), an opioid-dependent error signal also underlies second-order fear conditioning and sensory preconditioning. This, in turn, raises two immediate questions: what is learned in second-order conditioning and sensory preconditioning; and how is this learning regulated by error? An obvious possibility is that, in both cases: (1) animals learn to predict S1 when S2 is present and the error in this prediction drives formation of an S2–S1 association; and (2) test presentations of the S2 then retrieve this association, which is “chained” with the S1-shock association to generate fear to the S2. However, the available evidence suggests that this rarely occurs in protocols of the sort used in this study (forward serial pairings of a visual and auditory stimulus); and two aspects of the present findings suggest that this was not the case here. In Experiments 1b and 2b, naloxone enhanced first-order fear conditioning to S1 but this did not automatically increase second-order or sensory preconditioned fear to S2, as predicted by the chaining account: e.g., rats injected with vehicle prior to the S2–S1 pairings exhibited the same test level of freezing to S2 regardless of whether they had been injected with naloxone or vehicle prior to the S1-shock pairings. Therefore, we take these findings to mean that second-order conditioning and sensory preconditioning are not due to chaining of the S2–S1 and S1-shock associations at the time of testing with S2; and by extension, that the naloxone-induced enhancements of second-order conditioning and sensory preconditioning reflect a broader role for prediction error in different types of associative formation.

What then is learned in second-order conditioning and sensory preconditioning; and how is this learning affected by naloxone? The available evidence suggests that, in protocols like the ones used here, the learning that underlies second-order and sensory preconditioned fear is not the same. When S2 is paired with S1 in second-order conditioning, it associates with the central state of fear elicited by the S1: i.e., animals form an S2-fear association that exists independently of the already-conditioned S1-shock association (for further discussion, see Rizley and Rescorla, 1972; Rescorla, 1973, 1982; Holmes et al., 2014). In contrast, when S2 is paired with a neutral S1 in sensory preconditioning, animals *do* form an S2–S1 association; but this is not chained with the S1-shock to generate fear of S2 at testing. Rather, when S1 is conditioned in stage 2, it calls to mind its past associate, the S2, and thereby, mediates an association between the memory of S2 and the foot shock US (Holland, 1981; for supporting data, see Wong et al., 2019). Therefore, we take the present findings to mean that prediction error differentially regulates the S2-fear, S2–S1, and mediated S2-shock associations that form in second-order conditioning and sensory preconditioning. Naloxone preserves error in relation to the S2 and fear, thereby enhancing second-order fear conditioning across the S2–S1 pairings. Similarly, naloxone preserves error in relation to the S2 and S1 events in sensory preconditioning, resulting in stronger S2–S1 associative formation in stage 1, and thereby, retrieval-mediated conditioning of S2 in stage 2. In contrast, naloxone does not affect the mediated S2-shock association that forms when animals are exposed to S1-shock pairings in sensory preconditioning, suggesting that this association is not regulated by prediction error. We propose that the mediated S2-shock association differs from the others in this respect because it involves learning about a retrieved stimulus representation, which may be governed by a different set of rules (e.g., Bae et al., 2015; Lingawi et al., 2018). This hypothesis will be tested in future studies.

Finally, the opioid-dependent error signal that underlies Pavlovian conditioning was inferred from the contrasting effects of naloxone on the acquisition and extinction of first-order fear (McNally, 2009); and has been identified with activity in midbrain circuits including the amygdala and periaqueductal gray (McNally and Cole, 2006; Johansen et al., 2010; Yau and McNally, 2018). At present, the effects of naloxone on extinction of second-order and sensory preconditioned fear are unknown, as are the neural substrates of its effects on second-order conditioning and sensory preconditioning more generally. However, it seems reasonable to predict that naloxone will impair extinction of second-order and sensory preconditioned fear in the same way as it has been shown to impair extinction of first-order fear; and further, that the neural substrates of its effects on second-order fear conditioning and sensory preconditioning will involve the same regions that have been shown to regulate its effects on first-order fear conditioning and its extinction. Specifically, given the critical involvement of the basolateral amygdala complex (BLA) in acquisition and extinction of first-order, second-order and sensory preconditioned fear (Gewirtz and Davis, 1997; Parkes and Westbrook, 2010; Holmes et al., 2013, 2018; Lay et al., 2018; Lingawi et al., 2021), it is likely that an opioid-dependent error signal regulates associative formation in each of these cases via its effects in this region of the brain.

In summary, the present series of experiments has shown that the endogenous opioid system regulates associative formation whenever two events are paired and independently of their affective content. They thus confirm that endogenous opioids do not only affect US processing in Pavlovian fear conditioning: they also encode an error signal that reflects the discrepancy between observed and expected events. Endogenous opioids do not, however, regulate conditioning to a retrieved stimulus representation, presumably because it occurs independently of prediction error. Future work will test this hypothesis, the effects of naloxone on extinction of second-order and sensory preconditioned fear, and finally, the neural substrates of these effects in the BLA. Specifically, it will examine whether naloxone impairs extinction of second-order and sensory preconditioned fear in the same way as it has been shown to impair the extinction of first-order fear and other forms of learning produced by CS alone exposure (e.g., latent inhibition; Leung et al., 2013); and whether naloxone achieves its effects on second-order conditioning and sensory preconditioning via its effects in the BLA.

## Data Availability Statement

The raw data supporting the conclusions of this article will be made available by the authors, without undue reservation.

## Ethics Statement

The animal study was reviewed and approved by the University of New South Wales Animal Care and Ethics Committee.

## Author Contributions

NH and RW designed the study. RM and DL conducted the experiments, collected, and analyzed the data. RM, NH, and RW wrote the manuscript. All authors contributed to the article and approved the submitted version.

## Conflict of Interest

The authors declare that the research was conducted in the absence of any commercial or financial relationships that could be construed as a potential conflict of interest.

## Publisher’s Note

All claims expressed in this article are solely those of the authors and do not necessarily represent those of their affiliated organizations, or those of the publisher, the editors and the reviewers. Any product that may be evaluated in this article, or claim that may be made by its manufacturer, is not guaranteed or endorsed by the publisher.
